# Supplementary material for: Does adding MRI and CSF-based biomarkers improve cognitive status classification based on cognitive performance questionnaires?
Source: PLoS One. 2023 May 8;18(5):e0285220. doi: 10.1371/journal.pone.0285220 (PMC10166486; doi:10.1371/journal.pone.0285220)
Supplement: S1 Table — (DOCX) [file pone.0285220.s001.docx]

| **S1 Table. Specificity and Sensitivity of Cognitive Status Classification Model with MMSE only and MMSE + MRI + CSF Predicting Diagnosed Cognitive Status** | | | | | |
| --- | --- | --- | --- | --- | --- |
| **Panel A. Model with Demographics + MMSE Only (Model 2)** | | | |  |  |
|  | **Diagnosed Cognitive Status** | | |  |  |
| **Predicted Cognitive Status** | **Cognitive Normal** | **CIND** | **Dementia** |  |  |
| Cognitively Normal | 35 | 33 | 0 |  |  |
| Cognitive Impairment without Dementia (CIND) | 122 | 437 | 53 |  |  |
| Dementia | 1 | 34 | 117 |  |  |
| **Percent Correctly Predicted** | 22% | 87% | 69% | **Overall Correctly Predicted :** | 71% |
|  |  |  |  |  |  |
| **Panel B. Model with MMSE + MRI + CSF (Model 5)** | | | |  |  |
|  | **Diagnosed Cognitive Status** | | |  |  |
|  | **Cognitive Normal** | **CIND** | **Dementia** |  |  |
| Cognitively Normal | 55 | 39 | 0 |  |  |
| Cognitive Impairment without Dementia (CIND) | 102 | 433 | 51 |  |  |
| Dementia | 1 | 32 | 119 |  |  |
| **Percent Correctly Predicted** | 35% | 86% | 70% | **Overall Correctly Predicted :** | 73% |
