# Supplementary material for: Does adding MRI and CSF-based biomarkers improve cognitive status classification based on cognitive performance questionnaires?
Source: PLoS One. 2023 May 8;18(5):e0285220. doi: 10.1371/journal.pone.0285220 (PMC10166486; doi:10.1371/journal.pone.0285220)
Supplement: S2 Table — (DOCX) [file pone.0285220.s002.docx]

| **S2 Table. Multinomial Regression Model Predicting Cognitive Status at Baseline with Plasma Marks of P-Tau and Aß** | | | | | | |
| --- | --- | --- | --- | --- | --- | --- |
|  | Plasma Ptau |  | Plasma Aß40 |  | Plasma Aß42 |  |
| **Dementia** |  |  |  |  |  |  |
| Age | 0.0130 | [0.0233] | -0.00581 | [0.0285] | -0.00744 | [0.0287] |
| Male | 0.483 | [0.331] | -0.0129 | [0.395] | -0.0157 | [0.397] |
| Education (Years) | -0.149* | [0.0629] | -0.0642 | [0.0684] | -0.0592 | [0.0686] |
| APOE4 Alleles |  |  |  |  |  |  |
| 1 Allele | 1.769*** | [0.370] | 1.590*** | [0.412] | 1.623*** | [0.415] |
| 2 Alleles | 2.163*** | [0.559] | 3.901*** | [1.081] | 3.912*** | [1.082] |
| Ptau Plasma | 0.0174 | [0.0848] |  |  |  |  |
| Plasma Aß 40 |  |  | 0.0181 | [0.0165] |  |  |
| Plasma Aß 42 |  |  |  |  | 0.00472 | [0.00357] |
| Constant | 0.440 | [2.111] | -0.280 | [2.397] | -0.303 | [2.404] |
|  |  |  |  |  |  |  |
| **CIND** |  |  |  |  |  |  |
| Age | -0.0272 | [0.0184] | -0.0402 | [0.0232] | -0.0464* | [0.0235] |
| Male | 0.295 | [0.262] | 0.715* | [0.339] | 0.689* | [0.341] |
| Education (Years) | -0.0758 | [0.0515] | 0.000445 | [0.0590] | -0.00146 | [0.0591] |
| APOE4 Alleles |  |  |  |  |  |  |
| 1 Allele | 1.024*** | [0.302] | 1.214*** | [0.348] | 1.304*** | [0.351] |
| 2 Alleles | 0.909 | [0.509] | 2.533* | [1.061] | 2.646* | [1.063] |
| Plasma P-Tau | -0.229 | [0.161] |  |  |  |  |
| Plasma Aß 40 |  |  | -0.00637 | [0.0138] |  |  |
| Plasma Aß 42 |  |  |  |  | 0.00292 | [0.00301] |
| Constant | 4.171* | [1.690] | 2.730 | [1.975] | 2.547 | [1.981] |
| Observations | 532 |  | 252 |  | 252 |  |
| Pseudo R-squared | 0.061 |  | 0.103 |  | 0.102 |  |
|  |  |  |  |  |  |  |
| Source: ADNI data |  |  |  |  |  |  |
| * p<0.05, ** p<0.01, *** p<0.001 | | |  |  |  |  |
