# Supplementary material for: Does adding MRI and CSF-based biomarkers improve cognitive status classification based on cognitive performance questionnaires?
Source: PLoS One. 2023 May 8;18(5):e0285220. doi: 10.1371/journal.pone.0285220 (PMC10166486; doi:10.1371/journal.pone.0285220)
Supplement: Attachment — Submitted filename: response_reviewer_final.docx [file pone.0285220.s003.docx]

We thank the editor and reviewers for the opportunity to improve the manuscript. We were pleased with the overall positive assessment of the manuscript, and in response to the reviewers’ comments, we have amended manuscript to further clarify our contribution and have highlighted the importance of MRI and CSF biomarkers for research on brain pathology. We believe that the reviewers have helped us improve our contribution. Our response to each point and manuscript edits are detailed below:

Reviewer #1: Authors reported, MRI and CSF biomarkers, while important for understanding dementia pathology in clinical research, were not found to substantially improve cognitive status classification based on cognitive status performance, which may limit adoption in population-based surveys due to costs, training, and invasiveness associated with their collection.

This is well conducted study.

We thank the reviewer for their consideration of the manuscript and appreciate their positive remarks.

Reviewer #2: As imaging, cerebrospinal fluid, and blood biomarkers are achieving more attention in the diagnosis of dementia, this is an important study that reaffirms the importance of assessing cognitive performance in the diagnosis of dementia and cognitive impairment. However, I have a major concern.

Authors mention that MRI and CSF biomarkers, while important for understanding dementia pathology in clinical research, were not found to substantially improve cognitive status classification based on cognitive status performance. First of all, isn't that a given? Unless, overuse of biomarkers would interfere with classification of cognitive status, imaging and CSF biomarkers are necessary to investigate the "causes" of cognitive impairment, not to detect the "presence" of cognitive impairment. For example, the presence of Alzheimer's pathology in the brain does not necessarily result in Alzheimer's dementia. Without cognitive performance tests and assessment of ADLs, it is impossible to determine if dementia is present in Alzheimer's disease. Even in the diagnostic criteria for Alzheimer's disease dementia, no imaging or CSF biomarkers are needed to diagnose “probable AD dementia”. On the other hand, once the cognitive impairment is observed, imaging, CSF, and blood biomarkers are useful to determine whether it is due to the accumulation of specific proteins, or associated with cerebrovascular disease, or whether it is a treatable dementia such as iNPH and epilepsy.

Secondly, authors conclude that MRI and CSF biomarkers, which may limit adoption in population-based surveys due to the cost, training, and invasiveness associated with their collection. However, for example, this study did not examine the cost-effectiveness of using imaging and CSF biomarkers in population-based surveys. Understanding the underlying pathology of dementia for the appropriate diagnosis and medication, such as use of disease-modifying drugs, may allow patients to stay in their homes longer and reduce total health care costs. Furthermore, reviewer disagree with the indiscriminate implementation of invasive testing. However, future search for non-invasive and inexpensive biomarkers will have greater benefits, but for development, comparisons with currently established invasive biomarkers are needed.

We thank the reviewer for the comments that highlight the importance of biomarkers for research, especially as it pertains to pathology. To address the first concern the reviewer brings up about whether it is a given that dementia pathology markers do not improve cognitive status classification based on cognitive performance, we believe that this area of debate remains open. Scientific surveys, especially large nationally representative studies, are trying to determine how best to improve cognitive status classification, especially as it becomes an increasingly important area of research. Ongoing debates are being held about the influence of practice effects and cultural differences that impact exam results using current practices in surveys (Feeney et al., 2016; Gianattasio et al., 2019; Hale et al., 2020). Also, there is room for cognitive status classification to improve. Some of the best performing measures correctly classify respondents have a sensitivity that ranged from 18% to 62%, indicating there is remove from improvement in correctly classifying people with cognitive impairment (Gianattasio et al., 2019) . Therefore, we believe that these questions are not settled, and that this manuscript contributes to this conversation.

One area of significant debate is whether and how to incorporate brain pathology markers to improve cognitive status classification in addition to cognitive performance questionnaires. We agree with the reviewer that dementia classification necessitates the inclusion of poor cognitive functioning—the opposite has not been argued in the manuscript. In fact, our introduction and analytical approach centers on the importance of cognitive functioning markers by focusing on the use of cognitive functioning tests for classification of cognitive status in studies. We then add brain pathology biomarkers to observe whether the classification based on cognitive functioning is improved. We recognize that imaging, CSF, and blood-based biomarkers are important for understanding pathology. Our conclusion is not to discourage their collection but rather to clarify how best to use them and when to incorporate them in population-based studies and that if they are used for improvements of cognitive status classification then their application may be more limited. We do not believe that the positions are in opposition. To address the reviewer’s concerns, we have amended the manuscript to recognize the importance that research to improve understanding of the underlying pathological developments which put people at risk for dementia will have large and important for individuals, communities, and populations. We have added text to the manuscript as follows:

“Additionally, it is also important to note that while we find minimal improvements in cognitive status classification with the inclusion of MRI and CSF biomarkers, these markers may be more important for other types of scientific questions that will push cognitive health research forward. For example, understanding the underlying pathology for cognitive impairment in the population may provide greater insight into potential treatments that will improve the health and wellbeing of individuals and can have implications for health inequities. Contributions from these types of studies cannot be understated. Therefore, in considering whether to add these MRI and CSF markers to population-based surveys that often collect information on several thousand individuals, researchers will have to determine the purpose and use of the markers beyond cognitive status classification, whether population-based samples can add to scientific insight beyond existent or emerging community or clinical-based studies, and the extent to which their studies will participate (such as a targeted group or randomized subsample).” (pg.8)

Reviewer #3: This paper systematically investigated the classification of Cognitive status (Dementia, cognitive impairment without dementia and normal) using multinomial logistic regression. The regression is performed with different models on the basis of combination of covariates. The study is primarily focused on the contribution of MRI and CSF biomarkers to classification of cognitive status.
I have some concerns on this paper.
1) The paper is well written and the finding of the paper is clearly described.
2) The classification is done on the basis of odd ratio using multinomial logistic regression. Results could be presented graphically so that the patterns, trends and relationships between different variables could be easily understood.

We thank the reviewer for this suggestion. We have debated and considered how best to incorporate reviewer feedback as it pertains to Table 2. However, we have not found a simple solution that would not lead to potential confusion. Usually, graphical representations of coefficient changes are used in model series that add additional covariates in a subsequent manner to determine how specific coefficients change based on the inclusion. In this manuscript, the emphasis has been placed more or lesson on the changes in explanatory power of each model as characterized by the R^2^ and covariates are not added subsequentially. Therefore, we believe that a graphical representation may obfuscate the main purpose of the models.

3) Numerous papers are published on Cognitive status classification using the biomarkers (MRI, CSF) used in this paper. Only the difference is, majority of works classify on the basis of probability and the proposed method classified based on odd ratio. What is the major contribution in this paper besides expressing the classification in terms of odds ratio?

We thank the reviewer for the question about the contribution. We have amended the manuscript to highlight our contribution so that it will remain clear for future readers. Briefly, our contribution is not the use of odds ratios to predict cognitive status classification. Rather the contribution is considering whether cognitive status classification based on cognitive functioning tests improves with additional MRI and CSF biomarkers. To the best of knowledge, this question has not been addressed before. Instead, research has largely observed them separately, considering their individual associations with cognitive impairment.

Feeney, J., Savva, G. M., O’Regan, C., King-Kallimanis, B., Cronin, H., & Kenny, R. A. (2016). Measurement Error, Reliability, and Minimum Detectable Change in the Mini-Mental State Examination, Montreal Cognitive Assessment, and Color Trails Test among Community Living Middle-Aged and Older Adults. *Journal of Alzheimer’s Disease: JAD*, *53*(3), 1107–1114. https://doi.org/10.3233/JAD-160248

Gianattasio, K. Z., Wu, Q., Glymour, M. M., & Power, M. C. (2019). Comparison of Methods for Algorithmic Classification of Dementia Status in the Health and Retirement Study. *Epidemiology (Cambridge, Mass.)*, *30*(2), 291–302. https://doi.org/10.1097/EDE.0000000000000945

Hale, J. M., Schneider, D. C., Gampe, J., Mehta, N. K., & Myrskylä, M. (2020). Trends in the Risk of Cognitive Impairment in the United States, 1996–2014. *Epidemiology (Cambridge, Mass.)*, *31*(5), 745–754. https://doi.org/10.1097/EDE.0000000000001219
